# Toxicity of Crude Oil Wastewater Treated with Nano-ZnO as a Photocatalyst on *Labeo rohita*: A Biochemical and Physiological Investigation

**DOI:** 10.3390/jox15010025

**Published:** 2025-02-02

**Authors:** Zahra Mousaviyon, Hamid Reza Pourkhabbaz, Mahdi Banaee, Saeid Khodadoust, Ali Reza Pourkhabbaz, Abha Trivedi, Caterina Faggio, Cristiana Roberta Multisanti

**Affiliations:** 1 Environmental Department, Faculty of Natural Resources, Behbahan Khatam Alanbia University of Technology, Behbahan 1417614411, Iran; mousaviyon93@gmail.com (Z.M.); pourkhabbaz@yahoo.com (H.R.P.); 2 Aquaculture Department, Faculty of Natural Resources, Behbahan Khatam Alanbia University of Technology, Behbahan 6361647189, Iran; 3 Department of Chemistry, Behbahan Khatam Alanbia University of Technology, Behbahan 6361647189, Iran; saeid.kh64@gmail.com; 4 Department of Environmental Sciences, Faculty of Natural Resources and Environment, University of Birjand, Birjand 9717434765, Iran; apourkhabbaz@yahoo.com; 5 Toxicogenomics Laboratory, Department of Animal Science, M.J.P. Rohilkhand University, Bareilly 243006, India; abha14sep@gmail.com; 6 Department of Chemical, Biological, Pharmaceutical and Environmental Sciences, University of Messina, 98166 Messina, Italy; 7 Department of Eco-sustainable Marine Biotechnology, Stazione Zoologica Anton Dohrn, 80122 Naples, Italy; 8 Department of Veterinary Sciences, University of Messina, Viale Giovanni Palatuccisnc, 98168 Messina, Italy; cristiana.multisanti@studenti.unime.it

**Keywords:** freshwater fish, crude oil wastewater, nano-ZnO, biomonitoring, bio-indicators

## Abstract

This study aimed to evaluate the effects of the water-soluble fraction of crude oil (WSFO) on Indian carp (*Labeo rohita*) with and without treatment with zinc oxide nanoparticles (Nano-ZnO). A total of 225 fish were randomly assigned to five groups in triplicate for 21 days. Group I served as the control group. Groups II and III were exposed to 0.5% and 1% untreated WSFO, respectively. Groups IV and V received 5% and 10% WSFO treated with Nano-ZnO, while Groups VI and VII received 5% and 10% WSFO treated without Nano-ZnO. No blood samples were obtained from fish exposed to untreated WSFO, due to increased hemolysis. Exposure to treated WSFO increased creatine phosphokinase, alkaline phosphatase, aspartate aminotransferase, lactate dehydrogenase, and gamma-glutamyl transferase activities, while alanine aminotransferase activity decreased. Although a significant decrease was observed in total protein, globulin, and triglyceride levels, albumin and cholesterol increased. Thiol groups and glutathione peroxidase activity significantly decreased, while superoxide dismutase, catalase, total antioxidant capacity, and malondialdehyde levels increased. The findings showed that exposure to WSFO, whether treated or untreated, induces significant biochemical and oxidative stress responses in *Labeo rohita*. Although WSFO treated with Nano-ZnO mitigated hemolysis, it was unable to prevent enzyme and antioxidant imbalances, indicating persistent physiological stress.

## 1. Introduction

The contamination of aquatic ecosystems represents a significant and growing contemporary issue [1,2,3,4,5,6,7]. In particular, light crude oil presents significant environmental and toxicological concerns due to its low density and high content of volatile organic compounds (VOCs), including benzene, toluene, ethylbenzene, and xylene (BTEX), as well as polycyclic aromatic (PAHs) [8,9,10,11,12,13,14]. These compounds are highly soluble and bioavailable, thereby increasing their hazardous effects on aquatic environments [15,16,17,18,19].

The contamination of light crude oil in freshwater ecosystems often originates from spills during transportation (pipelines, rail, or tankers), accidents at drilling sites, and discharges of oil-contaminated wastewater from refineries. Spills and natural runoff from urban areas and industrial facilities can also contribute. The lighter fraction of crude oil disperses rapidly, increasing its spread in aquatic systems. Hydrocarbon pollution, even in small volumes, can influence aquatic environments. Kharey et al. [20] conducted a mesocosm study simulating a crude oil spill in a pristine freshwater ecosystem in Canada. Massod et al. [21] evaluated petroleum contamination in the sediments of the Selangor River, reporting PAH levels ranging from 219.7 to 672.3 ng/g, with a maximum concentration of 964.7 ng /g. Sharifi et al. [13] found moderate to PAH pollution in Brunei Bay sediments (826.7 to 2955.3 μg/kg). Once introduced, light crude oil may coat surfaces such as sediments, aquatic vegetation, and organisms, disrupting photosynthesis and gas exchange. In addition, volatile fractions of light crude oil can dissolve in water and cause acute and chronic toxicity to aquatic life [22]. Toxic hydrocarbons can bioaccumulate in organisms, especially fish and invertebrates, and magnify up the food chain, posing risks to higher trophic levels, including humans. Crude oil spills into aquatic ecosystems can significantly impact the physicochemical quality of water and nutrient cycling. Studies have shown that petroleum pollution can devastate freshwater habitats such as wetlands, which serve as critical breeding sites and centers of biodiversity. Biodiversity loss and changes in ecological interactions are among the most critical environmental concerns caused by increased oil pollution [17,23,24,25]. The toxic effects of crude oil and its derivatives, such as polycyclic aromatic hydrocarbons (PAHs), are complex and depend on exposure levels, duration, and species susceptibility [26]. Nwizugbo et al. [27] reported that African sharptooth catfish (*Clarias gariepinus*) exposed to light crude oil showed elevated biomarkers (LDH, GLU, G-6-PDH, DNA, RNA) and metabolic stress, with more significant effects on wild populations compared to laboratory conditions. Similarly, Liao et al. [28] found reduced growth, lipid content, and oxidative damage in tiger puffer (*Takifugu rubripes*) exposed to dietary hydrocarbons, with incomplete recovery after 4 weeks. Histopathological damage, including liver cell swelling and kidney glomerular occlusion, was observed in *Rutilus frisii* exposed to the WSF of crude oil, with effects intensifying at higher concentrations [29]. Ecotoxicological assessments by Zheng et al. [14] using *Oryzias melastigma* embryos revealed variable stress levels linked to PAHs and petroleum hydrocarbons, emphasizing the need for advanced monitoring tools like OME-FES.

Scientists are continually working to minimize oil pollution in aquatic ecosystems and its environmental consequences using various methods [24]. One proposed solution is using nanotechnology to remove oil pollution and treat wastewater from the oil industry [30]. Although nanoparticles are often investigated for their toxic effects [15,31,32,33], using zinc oxide nanoparticles (Nano-ZnO) is a highly effective photocatalyst method for industrial wastewater treatment due to their excellent photocatalytic properties under UV or sunlight. With a wide band gap (~3.37 eV) and high exciton binding energy (~60 meV), Nano-ZnO can degrade a wide range of organic pollutants [34]. When exposed to UV light, ZnO absorbs photons and produces electron–hole pairs, which lead to the formation of reactive oxygen species (ROS), such as hydroxyl radicals (•OH) and superoxide anions (O2−) [15,32,35,36]. These ROS attack complex pollutants, including dyes, phenols, pharmaceuticals, and hydrocarbons, and degrade them into non-toxic end products such as CO_2_ and H_2_O. Zinc oxide nanoparticles offer several advantages, including high photocatalytic efficiency due to their large surface area, broad-spectrum activity, chemical stability, cost-effectiveness, and environmental friendliness. However, challenges exist, such as UV dependence, nanoparticle aggregation, secondary pollution, and recovery issues. Recent studies highlight the versatility of Nano-ZnO in addressing oil-related environmental challenges. Alsharyani and Muruganandam [30] demonstrated the efficient photocatalytic degradation of docosane in oil-produced water using ZnO nanorods, achieving 68.5% removal and 60.5% TOC reduction under solar light. Similarly, Baig et al. [15] showcased ZnO-coated Al_2_O_3_ membranes for oil–water separation, achieving a 99.66% separation efficiency and self-cleaning via UV-induced photocatalysis. Moreover, for enhanced oil recovery (EOR), Ahmadi et al. [31] reported that ZnO-Ce nanocomposites improved wettability and interfacial tension, achieving a 71.4% recovery factor. Soleimani et al. [37] optimized ZnO nanofluids for surface tension reduction, resulting in an 11.82% recovery efficiency. Furthermore, Ehmedan et al. [38] combined ZnO and Fe_2_O_3_ nanoparticles with bacteria to accelerate crude oil biodegradation, achieving over a 96% efficiency in three days. Yashni et al. [39,40] investigated the use of biosynthesized ZnO-NPs from orange peel extract for treating textile dye wastewater. Most of these studies emphasized the potential of ZnO nanoparticles in cleaning up environments contaminated with crude oil and its derivatives. Therefore, the use of nanotechnology can be a practical solution to reduce the level of oil pollutants in the environment.

However, a challenge remains, namely evaluating the efficiency of using Nano-ZnO to clean environments contaminated with crude oil and its derivatives. There are various strategies and solutions for monitoring environmental pollution, such as the use of bio-indicator organisms. The efficiency, flexibility, and cost of using bio-indicators have made this method one of the most common methods in environmental toxicology. This approach would be the sensitivity of aquatic organisms to environmental changes and allow the detection of toxicological effects at sub-lethal levels of pollutants [2,41]. Compared to direct chemical analysis, which often requires complex instrumentation to detect pollutants at very low concentrations, the biomarker-based approach is more cost-effective, accessible, and applicable for routine monitoring. Therefore, focusing on the physiological and biochemical responses of fish exposed to pollutants reduces the reliance on expensive and resource-intensive techniques such as high-performance liquid chromatography (HPLC) or mass spectrometry. Moreover, this alternative method provides valuable insights into the ecological health of water bodies by evaluating the biological responses of these organisms to various contaminants. In contrast to traditional chemical assays, assessing these biomarkers offers a deeper insight into contaminants’ biological and physiological effects [15,42,43]. Biological monitoring is ecologically meaningful and cost-efficient, providing a general evaluation of environmental quality while minimizing the dependence on elaborate laboratory procedures [33].

Bio-indicator animals, such as fish, crustaceans, and molluscs, can serve as valuable tools for assessing water pollution by reflecting the presence of contaminants through changes in their different biomarkers [2,41,44,45]. The studies showed that these biomarkers can be an indirect and efficient alternative to traditional methods of measuring physicochemical parameters in water and wastewater. These biomarkers, including enzyme activities, antioxidant markers, and other biochemical parameters, serve as sensitive biomarkers of exposure to xenobiotics pollutants such as heavy metals, petroleum hydrocarbons, pesticides, microplastics, etc. [28,29,30,31,32,33]. Fuller et al. [46] evaluated the toxicity of dispersant-treated and untreated crude oil using different fish and shrimp species. A significant increase in mortality rates was reported in juvenile sea bass after exposure to dispersed crude oil and petroleum hydrocarbons [47]. Moreover, Greer et al. [48], Greer et al. [49], Adams et al. [50], and Gissi et al. [51] found that exposure to dispersed oil solutions could increase mortality and deformities in fish embryos. Similarly, Wu et al. [52] found that chemically dispersed crude oil exhibited increased toxicity due to enhanced hydrocarbon partitioning into water. Cardiotoxicity and a change in biotransformation enzymes were observed in zebrafish and sheepshead minnows exposed to crude oil [53]. Following crude oil exposure, Khursigara et al. [54] reported reduced growth and metabolic rates in red drum (*Sciaenops ocellatus*). Leggieri et al. [55] observed increased CYP1A activity in the gills and liver of rainbow trout exposed to crude oil.

This study aimed to evaluate the potential of Indian carp (*Labeo rohita*) as a bio-indicator species for the monitoring of crude oil pollution in freshwater ecosystems. *L. rohita* was selected due to its ecological and economic significance in aquaculture, making it a relevant model for studying the impacts of environmental stressors. Moreover, the biochemical and physiological responses observed in this species can provide valuable insights into general mechanisms of aquatic toxicology, which may have implications for other species with similar ecological niches. It was hypothesized that exposure to light crude oil would induce significant changes in key biomarkers of *L. rohita*, reflecting the physiological stress and toxicity of the pollutant. Furthermore, it was hypothesized that the use of nano-zinc oxide as a photocatalytic modification agent would help reduce these adverse effects.

## 2. Materials and Methods

**Figure 1:**
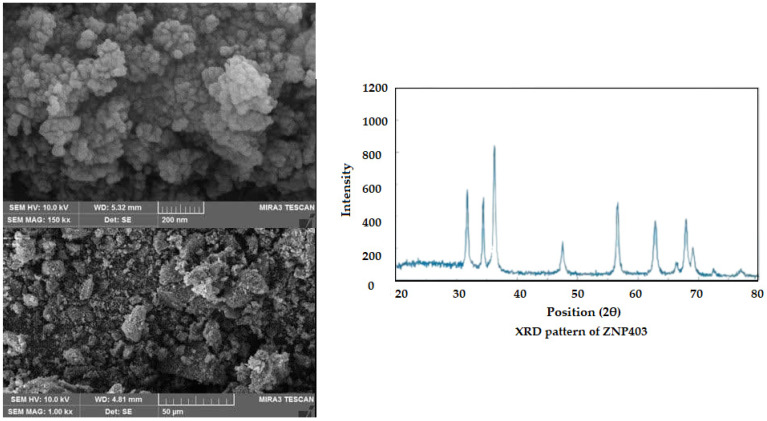
Properties of nanoparticles of ZnO (adopted from the brochure of Armina Engineering Company, Iran). Scanning electron microscopy (TESCAN-Vega3; Brno, Czech Republic) and X-ray diffraction (D8 ADVANCE; Bruker, Karlsruhe, Germany).

Nonionic surfactant Triton X-100 (Sigma-Aldrich, St. Louis, MO, USA), sodium dodecyl sulfate (SDS) (Sigma-Aldrich), identified crude oil (National Iranian Oil Company (NIOC), Tehran, Iran) (Table 1), biochemical diagnostic kits (ParsAzmun Company, Karaj, Iran), and nanoparticles of ZnO (Armina Engineering Company, Tehran, Iran) (Figure 1 and Table 2) were used in this study. The water used in all experiments was double-distilled water.

### 2.1. Preparation of Water-Soluble Fraction of Crude Oil (WSFO)

The water-soluble fraction of crude oil (WSFO) was prepared by mixing light oil at a concentration of 59.55 mg with 500 mL of distilled water. Next, 1 g of sodium dodecyl sulfate (SDS) was added as a surfactant to facilitate emulsification, and Tween 80 was used as an emulsion stabilizer to prevent phase separation. The mixture was heated to a temperature between 30 and 50 °C to facilitate better mixing with water. Then, a magnetic stirrer was employed to ensure thorough mixing of the components, resulting in a stable emulsion. The oil was dispersed uniformly in the water phase, creating a milky or cloudy mixture, indicating successful emulsification [33].

### 2.2. Treatment of WSFO

#### 2.2.1. Treatment with Gravity Separation Method

Distilled water was added to WSFO in a beaker and placed in an ultrasonic device (XUBA3) for 25 min to ensure thorough mixing. Following this step, the beaker was placed on a shaker (IKA) and exposed to artificial light from a linear fluorescent lamp (28 W; 2900 lumens; 104 lm/W) for 24 h to facilitate the interaction between the components. The solution was then centrifuged at 6000 rpm for 15 min to separate the oil from the water effectively. After centrifugation, the oil collected at the top was carefully separated, while the remaining milky water at the bottom of the Falcon tube was retained. The steps involving the addition of SDS as a surfactant and Tween 80 as an emulsion stabilizer were carefully repeated, followed by ultrasonication to ensure uniform dispersion of the mixture. Subsequently, the mixture underwent light treatment to promote the desired interactions and reactions, and centrifugation was performed at 6000 rpm for 15 min to achieve efficient separation of the components. These steps were repeated three more times to ensure consistency and thorough sample processing. In the final step, the pH of the resulting milky solution was adjusted to 5 by adding HCl. The sample was then centrifuged at 10,000 rpm for 15 min to obtain the final processed solution.

#### 2.2.2. Treatment with Nano-ZnO as Photocatalyst

An amount of 0.15 g of the Nano-ZnO was poured into distilled water and placed in an ultrasonic device (XUBA3) for 10 min. Afterward, it was added to the oil–water emulsion beaker and placed in the ultrasonic device for 25 min. After this stage, the beaker was placed on a shaker (IKA) and exposed to artificial light (linear fluorescent lamp (28 W; 2900 lumens; 104 lm/W)) for 24 h. The solution was placed in a centrifuge after blending the mixture for a suitable period. The centrifuge was set to 6000 rpm for 15 min to facilitate the sedimentation of Nano-ZnO, ensuring proper separation of unreacted particles. Following centrifugation, the oil collected at the top was carefully separated, while the milky water and nano-sediment at the bottom of the Falcon tube were retained. The steps involving the addition of Nano-ZnO, SDS as a surfactant, and Tween 80 as an emulsion stabilizer were carefully repeated, followed by ultrasonication to ensure uniform dispersion of the nanoparticles. Subsequently, the mixture underwent light treatment to achieve the desired interactions and reactions, and centrifugation was performed at 6000 rpm for 15 min to separate the components effectively. These steps were repeated three more times to ensure consistency and thorough sample processing. In the next step, the pH of the resulting milky solution was adjusted to 5. The sample was then centrifuged at 10,000 rpm for 15 min.

### 2.3. Analysis of Oil Concentration in Water

To determine the oil concentration in water at 230 nm, standard oil solutions (0.1–1.0 mg/mL) were prepared in hexane. The absorbance of the standards and the sample was measured at 230 nm after blanking the spectrophotometer with the solvent. A calibration curve (absorbance vs. concentration) was plotted, and the oil concentration in the sample was determined by comparing its absorbance to the curve.

### 2.4. Fish

In this study, 300 Indian carp (*Labeo rohita*) were obtained from an Aquatic breeding center in Ahvaz (Khuzestan Province, Iran) and transported to the Aquaculture Laboratory at the Faculty of Natural Resources, Khatam Alanbia University of Technology in Behbahan. Upon arrival, the fish were placed in suitable aquatic tanks and kept for a 2-week adaptation period under controlled conditions (temperature: 26 ± 2 °C, dissolved oxygen levels: 6.5 ± 0.5 mg/L, pH: 7.4 ± 0.2, and a photoperiod of 14 hours’ light and 10 hours’ dark). During this time, the water quality, including temperature, pH, and oxygen levels, was monitored to ensure optimal conditions for the fish.

### 2.5. Experimental Design

After the fish had been acclimatized and the treatment process had been completed, 225 Indian carp (*Labeo rohita*) were randomly distributed into fifteen 250 L tanks, with 15 fish per tank. Each tank was equipped with aerators to maintain proper oxygen levels, and 80% of the water was replaced every other day to ensure optimal water quality throughout the experiment (unionized ammonia (<0.05 mg/L), dissolved oxygen (6.5 ± 0.5 mg/L), temperature (26 ± 2 °C), and pH (7.4 ± 0.2)). This study was conducted with three replications and included seven experimental groups over 21 days. Group I was the control group, while Groups II and III consisted of fish exposed to 0.5 and 1% of untreated WSFO. The fish from group IV were exposed to a 5% concentration of WSFO treated with Nano-ZnO. Group V included fish exposed to a 10% concentration of WSFO treated with Nano-ZnO, Group VI consisted of fish exposed to a 5% concentration of WSFO treated without Nano-ZnO, and Group VII involved fish exposed to a 10% concentration of WSFO treated without Nano-ZnO. The fish was anesthetized with Clove powder (150 mg), and blood was sampled from *Labeo rohita* using a 2.5 mL syringe with heparin as an anticoagulant. The blood was transferred into a sterile blood collection tube containing heparin. The sample was centrifuged at 6000 rpm for 10–15 min at 4 °C. The plasma was carefully removed using a pipette, avoiding contamination with the buffy coat or red blood cells. The plasma was then stored at −25 °C for long-term storage or 4 °C for short-term use and was prepared for further analysis.

### 2.6. Blood Biochemical Parameters

Blood biochemical parameters (AST, ALT, ALP, GGT, CPK, LDH, glucose, cholesterol, triglycerides, albumin, total protein, and creatinine) were analyzed using assay kits purchased from Pars Azmoun Company. Plasma samples were prepared and transferred into sterile tubes for analysis. The Unico VIS/UV spectrophotometer (Model 2100, UNICO, Indianapolis, IN, USA) was used to measure absorbance. The assay reagents and standards were prepared according to the manufacturer’s instructions, and the spectrophotometer was calibrated using a blank reagent solution. The plasma samples were mixed with the appropriate reagents, incubated as specified in the kit protocols, and then analyzed for absorbance at the recommended wavelengths. Absorbance values were recorded, and the concentrations of each biochemical parameter were calculated using the calibration curves or standards. The globulin concentration was calculated by subtracting the albumin (ALB) concentration from the total protein (TP) concentration [56].

### 2.7. Statistical Analysis

Data from each experimental group were input into GraphPad Prism 10 (Graphpad Software, LA Jolla, CA, USA). The Shapiro–Wilk test was performed to assess the normality distribution of the data. One-way ANOVA was then conducted with a significance level of 0.05. Tukey’s test was used for pairwise comparisons, with *p*-values < 0.05 indicating statistical significance. The results are presented as mean ± standard deviation (SD).

## 3. Results

Changes in the blood biochemical parameters of fish exposed to treated crude oil wastewater are presented in Table 3. Blood samples from fish exposed to 0.5% and 1% WSFO were unsuitable due to severe erythrocyte hemolysis. As a result, valid data could not be collected. However, the hemolysis was not observed in the blood samples of fish exposed to WSFO treated with Nano-ZnO and WSFO treated without Nano-ZnO. These findings may be due to the reduced presence of xenobiotics following wastewater treatment, which significantly lowers toxicity.

Creatine phosphokinase (CPK), alkaline phosphatase (ALP), aspartate aminotransferase (AST), lactate dehydrogenase (LDH), and gamma-glutamyl transferase (GGT) activities significantly increased in all oil effluent treatments. However, alanine aminotransferase (ALT) activity decreased significantly after exposure to treated oil effluent compared with the control group. Although using Nano-ZnO could mitigate the toxicity of crude oil wastewater, significant changes were observed in all enzyme activities compared to the control group.

Although total protein and globulin levels in all treatments were significantly lower than in the control group, albumin levels were significantly increased after exposure to treated oil effluent. The results showed that whereas Nano-ZnO helped reduce the toxicity of crude oil wastewater, total protein, albumin, and globulin showed significant changes compared to the control group. Although cholesterol increased significantly in treatments, triglyceride levels dropped significantly in oil-effluent-treated groups. The highest cholesterol levels were observed in the plasma of fish exposed to 5% oil effluent treated without Nano-ZnO. However, the lowest triglyceride levels were found in the plasma of fish exposed to treated oil effluent without Nano-ZnO. The results showed that the treatment of crude oil wastewater with Nano-ZnO may have significantly affected changes in blood cholesterol and triglycerides in exposed fish. Glucose levels increased significantly in all treatments compared to the control. The highest levels were observed in the plasma of fish exposed to Nano-ZnO treatments.

**Figure 2:**
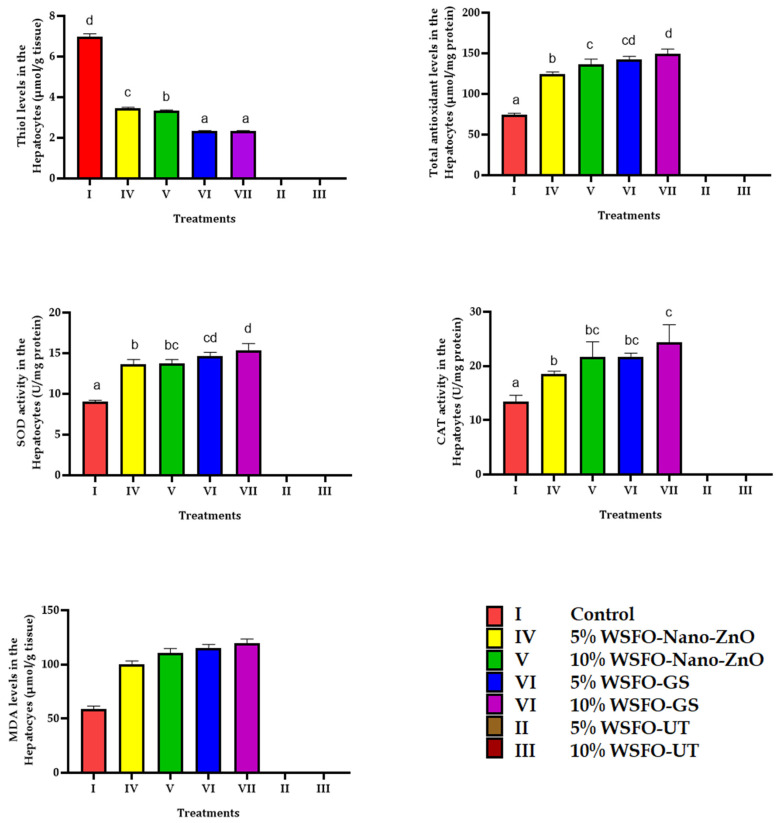
Changes in the oxidative biomarkers in the hepatocytes of fish. Different letters indicate the presence of significant differences, and similar letters indicate that there is no significant difference between different experimental groups. No data were obtained from group II (fish exposed to 5% untreated WSFO) and III (fish exposed to 10% untreated WSFO).

**Figure 3:**
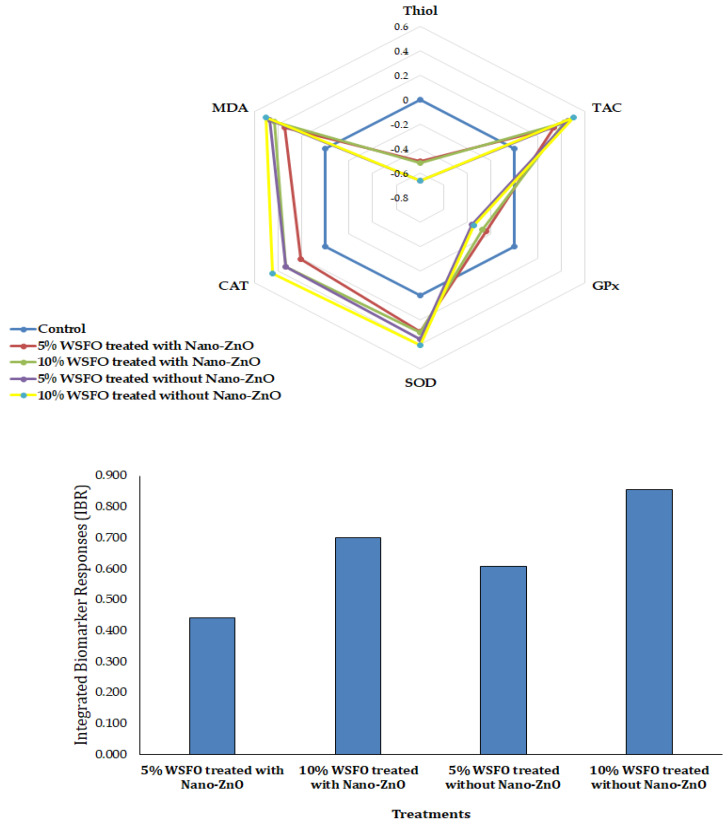
Alteration of the oxidative biomarkers in fish hepatocytes.

Changes in the oxidative biomarkers in the hepatocytes of fish are illustrated in Figure 2 and Figure 3. A significant decrease was observed in thiol group levels and GPx activity in the fish hepatocytes across all treatments compared with the control group. The findings suggest that using Nano-ZnO to treat oil effluent can effectively reduce xenobiotics in wastewater. A significant increase in the total antioxidant levels in the hepatocytes of fish exposed to crude oil wastewater indicated an active response of the antioxidant defense systems. Although the use of Nano-ZnO reduced the toxic effects of treated crude oil wastewater on TAC levels, these levels remained significantly higher than those in the control group. Exposure to treated crude oil wastewater resulted in a significant increase in SOD and CAT activities, as well as MDA levels, in the hepatocytes of fish. The highest SOD and CAT activities, along with the highest MDA levels, were observed in fish exposed to 10% oil effluent treated without the use of Nano-ZnO.

## 4. Discussion

Exposure to untreated WSFO led to an increased hemolysis rate in fish, and we could not assay the blood biochemical parameters and oxidative biomarkers. Therefore, we had to ignore these data. The findings indicated notable changes in blood biochemical parameters and oxidative stress biomarkers in fish exposed to treated WSFO. A significant increase in CPK, ALP, AST, LDH, and GGT activities could indicate cellular membrane damage. Xenobiotics and residual toxicants in WSFO may cause cell membrane damage and the leakage of cytoplasmic and transmembrane enzymes into the blood by inducing lipid peroxidation.

A notable elevation in plasma ALP activity in fish exposed to WSFO indicated the possibility of hepatobiliary dysfunction, liver damage, and associated tissue impairment. Furthermore, elevated ALP activity frequently indicated an adaptive response to cellular stress or injury, as the enzyme is released into the bloodstream due to the disruption of cell membranes in the liver, kidneys, intestine, or bones [57]. Moreover, a significant increase in ALP could also be indicative of metabolic disturbances triggered by the detoxification processes activated in response to the presence of toxic compounds [58]. Increased AST and LDH levels, particularly in higher treated WSFO concentrations without Nano-ZnO, confirmed hepatocellular injury and cytotoxicity [37,38]. Furthermore, the observed changes in LDH and GGT activities in fish plasma suggested that Nano-ZnO contributes to the detoxification of xenobiotics in treated WSFO. LDH and GGT are well-established biomarkers of cellular and hepatic stress, respectively, and their modulation indicated a reduction in the physiological burden imposed by hydrocarbon exposure [38]. However, a significant decrease in ALT activity indicated hepatoxicity effects [39]. Changes in these parameters might indicate the efficiency of Nano-ZnO in degrading some pollutants in SWFO. These results indicated that although ZnO nanoparticles could remove a considerable portion of xenobiotics in WSFO, metabolites and contaminant residues, even at very low concentrations over different periods, may significantly affect the health of aquatic animals and ecosystems. Karamati and Ayti [59] showed that the efficiencies of electrocoagulation (EC), photocatalysis with ZnO, and a combination of both methods in wastewater treatment and COD reduction under optimal conditions were 94, 76, and 85%, respectively, within 60–300 min. Guo et al. [32] and Kusworo et al. [60] found that using graphene oxide-ZnO nano-membranes can increase and reduce the oil removal efficiency and COD rate by 90–99% in industrial wastewater, respectively. Similar results were reported by Shaba et al. [36] and Kuang et al. [35], who use ZnO to treat wastewater.

Machanlou et al. [43] found that UV-treated WSFO caused significant toxicity in common carp (*Cyprinus carpio*), increasing ALT, AST, and ALP levels, while TiO_2_-NPs under UV reduced the toxicity of WSFO. Similarly, Gao et al. [42] reported that diesel oil-WSF altered biochemical markers in black rockfish (*Sebastes schlegelii*). In *Sebastes schlegelii*, water-soluble fractions (WSFs) of diesel oil caused decreased ALP and glucose levels, histological deformities in gills, and upregulated immune-related genes, indicating significant stress and immune activation [42]. Nwizugbo et al. [27] reported increased levels of LDH, glucose, glucose-6-phosphate dehydrogenase, total protein, and uric and nucleic acids (RNA, and DNA) in *Clarias gariepinus* exposed to light crude oil, indicating metabolic stress and developmental delays. A significant decrease was observed in acetylcholinesterase (AChE) activity in fish exposed to polycyclic aromatic hydrocarbons (PAHs) [61].

Changes in total protein, albumin, and globulin levels could reflect the physiological impact of xenobiotics in WSFO. Decreased total protein and globulin levels likely result from impaired protein synthesis due to hepatic stress, while elevated albumin levels may represent a compensatory response to maintain osmotic balance [62]. The significant decrease in total protein may be due to reduced amino acid absorption in the intestine of fish exposed to WSFO-GS. The findings showed that although WSFO with Nano-ZnO treatment might mitigate these disruptions, there were significant differences between WSFO treated with Nano-ZnO and the control group. Total protein and globulin levels significantly declined with WSFO exposure, particularly WSFO-GS, indicating impaired protein synthesis and immune suppression. The alterations in total protein, albumin, and globulin levels in fish exposed to Nano-ZnO-treated WSFO further emphasize the effectiveness of Nano-ZnO in pollutant remediation. Studies have shown these proteins are vital indicators of fish health and immune status. Normal total protein levels are a critical index of the proper physiological function of the liver, kidney system, and general health of fish [63]. Therefore, significant changes in their levels may indicate the efficiency of Nano-ZnO in degrading xenobiotics from WSFO. Moreover, these findings showed the potential of Nano-ZnO to degrade pollutants and minimize the immune and metabolic disruptions typically caused by WSFO.

Significant changes in cholesterol and triglycerides in fish exposed to SWFO treated with Nano-Zno and SWFO-GS indicated changes in lipid metabolism. Studies have shown that exposure to petroleum hydrocarbons could change lipid biosynthesis and catabolism in the liver of tiger puffer *Takifugu rubripes* [28]. Otitoloju & Olagoke [64] reported significant changes in the lipid profiles in *Clarias gariepinus* exposed to benzene and toluene. They showed that changes in cholesterol and triglycerides were related to oxidative damage in fish hepatocytes. Similar results were reported in the lipid profile of a *Danio rerio* embryo exposed to benzene [52]. Although no significant changes were observed in cholesterol levels in the plasma of fish exposed to WSFO treated with Nano-ZnO, their levels significantly increased in the fish exposed to WSFO-GS. These findings may indicate altered lipid metabolism from residual hydrocarbon exposure [65]. A significant decrease in triglyceride levels was observed in the plasma of fish exposed to WSFO-GS. These findings showed the role of Nano-ZnO in reducing the bioavailability of toxic compounds in WSFO that affect lipid metabolism. A significant increase in cholesterol and a decrease in triglycerides in the plasma of fish exposed to treated WSFO indicated changes in lipid metabolism due to stress or toxicity. Increased cholesterol levels may be an adaptive mechanism to oxidative stress to repair damage in cell membranes. Conversely, reduced triglyceride levels may indicate disrupted lipid storage or energy metabolism. Furthermore, the notable alterations in blood cholesterol and triglyceride levels observed in the treated groups suggest that Nano-ZnO may influence lipid metabolism, through its capacity to degrade hydrocarbon pollutants in wastewater [65,66].

Glucose levels increased significantly in all treatment groups, with the highest in Nano-ZnO-treated groups. This hyperglycemic response could indicate increased energy demand or a stress-induced mobilization of glycogen reserves. The significant increase in WSFO-treated fish showed that despite the removal of some xenobiotics in the Nano-ZnO-treated WSFO, glucose levels were significantly higher than in the control group. These findings suggested that the exposure of fish to WSFO-treated fish can lead to environmental stress. A significant increase in plasma glucose may reflect a physiological stress response. The hyperglycemia may be typically mediated by the activation of the hypothalamic–pituitary–inter-renal (HPI) axis, which leads to the release of cortisol, a key stress hormone. Cortisol promotes gluconeogenesis and glycogenolysis in the liver, increasing glucose availability to meet the heightened energy demands required to mitigate the toxic effects of xenobiotics and maintain homeostasis [63]. The blood biochemical changes, including elevated glucose and reduced total protein, ALT, and LDH activities, were reported in juvenile Beluga (*Huso huso*) following exposure to sub-lethal concentrations of crude oil for a long time [63].

Oxidative stress biomarkers in fish hepatocytes also displayed notable changes. Activities of SOD and CAT, along with MDA levels, increased significantly, indicating an upregulation of the antioxidant defense system to combat oxidative stress. The highest SOD and CAT activities, as well as MDA levels, were observed in fish exposed to 10% oil effluent treated without Nano-ZnO, reflecting the intense oxidative stress in these groups. The significant reduction in thiol group levels and GPx activity across all treatments indicates a depletion of antioxidant reserves due to oxidative damage [42]. Although Nano-ZnO-treated groups showed lower TAC levels than untreated groups, TAC levels remained significantly elevated compared to the control, suggesting an enhanced antioxidant response to counter oxidative stress. Hamidi et al. [33] and Sadauskas-Henrique et al. [23] reported that oxidative stress is a key reaction to hydrocarbon pollution. Significant changes in the activities of CAT, GPx, and SOD were observed in mrigal fish (*Cirrhinus cirrhosus*) and Amazonian species, along with elevated MDA levels, reflecting lipid peroxidation. Studies have shown that combined exposure to oil and microplastics in *Oreochromis niloticus* and *Lates calcarifer* increased oxidative stress, pro-inflammatory cytokine production, and histopathological damage, highlighting synergistic toxicity [67]. Increased lipid peroxidation and changed antioxidant enzymes were reported in the liver and muscles of the *Anabas testudineus* exposed to polycyclic aromatic hydrocarbons [68]. Oxidative stress was observed in *Astyanax altiparanae* and *Oreochromis niloticus* exposed to gasoline and engine oil, respectively [69,70].

## 5. Conclusions

Changes in the blood biochemical parameters and oxidative stress were measured in all fish exposed to treated WSFO-GS and WSFO-NanoZnO. The findings showed that treated WSFO could change some biomarkers. However, the blood hemolysis of the fish exposed to untreated WSFO did not permit us to measure these parameters. Therefore, these results indicated that the untreated WSFO effects on ecosystems and organisms that live in polluted sites could be very dangerous and deadly. Moreover, these findings showed that although using nanotechnology could have significant efficacy in removing oil pollution and decreasing some xenobiotics in WSFO, residual xenobiotics in treated WSFO may threaten the health of aquatic organisms. The high sensitivity of biomarkers to even minimal environmental contaminants reinforces this concern. These findings also showed that these biomarkers could be used to monitor water pollution.

## Figures and Tables

**Table 1:** Main properties of Iranian light crude oils (quoted from National Iranian Oil Co.).

Property	Typical Value	Unit	Property	Typical Value	Unit
API Gravity	33.5–34.0	degrees API	Nickel (Ni)	~2.0	ppm
Specific Gravity	0.849–0.850	-	Vanadium (V)	~4.5	ppm
Sulfur Content	1.18–1.22	% by weight	Conradson Carbon Residue (CCR)	~1.5	% by weight
Pour Point	−21 to −24	°C	Distillation Range	50–650+	°C
Flash Point	18–22	°C	Light Ends (C1–C4)	~20	%
Kinematic Viscosity (at 20 °C)	4.8–5.5	cSt	Middle Distillates (C5–C16)	~55–60	%
Total Acid Number (TAN)	0.05–0.10	mg KOH/g	Residual Fraction (C17+)	~20–25	%
Paraffin Content	~18–22	%	Hydrogen Content	~13.5	%
Wax Content	~10	%	Nitrogen Content	~0.06	%
Asphaltenes Content	~1.2	% by weight	Boiling Point Range	~30–620+	°C

**Table 2:** Properties of nanoparticles of ZnO (Armina Engineering Company, Iran).

Characterization	
CAS	1314-13-2
Stock No.	ZNP403
Molecular formula	ZnO
Molecular weight	81.38 (g/mol)
Form	Powder
Color	White
Morphology	Irregular (ZNP403)
Crystal structure	Hexagonal wurtzite
Size range	50–100 (nm)
Total impurity	99 (%)
Density (g/cm^3^)	5.606
Solubility	Insoluble

**Table 3:** Alterations in the blood biochemical parameters of fish exposed to treated crude oil wastewater.

	Control	5% WSFO Untreated	10% WSFO Untreated	5% WSFO Treated with Nano-ZnO	10% WSFO Treated with Nano-ZnO	5% WSFO Treated Without Nano-ZnO	10% WSFO Treated Without Nano-ZnO
CPK (U/L)	161.5 ± 20.2 ^a^	ND	ND	249.3 ± 58.1 ^bc^	213.6 ± 17.4 ^ab^	324.0 ± 60.9 ^d^	305.9 ± 79.2 ^cd^
ALP (U/L)	42.1 ± 3.3 ^a^	ND	ND	63.1 ± 11.5 ^c^	57.7 ± 5.8 ^bc^	60.3 ± 3.6 ^bc^	53.7 ± 5.8 ^b^
AST (U/L)	97.7 ± 5.8 ^a^	ND	ND	137.7 ± 24.6 ^ab^	170.3 ± 35.2 ^b^	142.7 ± 37.1 ^ab^	254.2 ± 105.6 ^c^
ALT (U/L)	34.4 ± 13.7 ^c^	ND	ND	11.9 ± 1.7 ^b^	5.3 ± 0.6 ^ab^	5.7 ± 1.9 ^ab^	2.9 ± 1.2 ^a^
LDH (U/L)	155.8 ± 25.9 ^a^	ND	ND	340.0 ± 81.9 ^bc^	265.8 ± 55.7 ^b^	378.8 ± 129.6 ^c^	383.0 ± 73.2 ^c^
GGT (U/L)	6.1 ± 1.0 ^a^	ND	ND	14.4 ± 4.4 ^c^	9.6 ± 1.2 ^b^	10.8 ± 1.3 ^b^	10.6 ± 1.1 ^b^
Total Protein (g/dL)	3.96 ± 0.87 ^c^	ND	ND	3.66 ± 0.98 ^bc^	2.81 ± 0.51 ^ab^	2.83 ± 0.70 ^ab^	2.54 ± 0.41 ^a^
Albumin (g/dL)	1.01 ± 0.11 ^b^	ND	ND	0.74 ± 0.22 ^a^	2.17 ± 0.07 ^d^	2.10 ± 0.16 ^d^	1.60 ± 0.35 ^c^
Globulins (g/dL)	2.95 ± 0.89 ^b^	ND	ND	2.92 ± 1.05 ^b^	0.64 ± 0.51 ^a^	0.73 ± 0.56 ^a^	0.94 ± 0.68 ^a^
Cholesterol (mg/L)	152.9 ± 7.3 ^a^	ND	ND	169.9 ± 12.9 ^ab^	184.2 ± 10.7 ^ab^	237.2 ± 51.8 ^c^	200.4 ± 20.7 ^b^
Triglycerides (mg/L)	248.0 ± 38.2 ^c^	ND	ND	192.5 ± 26.6 ^b^	181.3 ± 51.5 ^b^	87.3 ± 13.8 ^a^	94.6 ± 22.4 ^a^
Glucose (mg/L)	50.9 ± 8.2 ^a^	ND	ND	107.9 ± 12.5 ^c^	102.5 ± 14.1 ^c^	83.7 ± 6.8 ^b^	94.9 ± 11.4 ^bc^

Different letters indicate the presence of significant differences, and similar letters indicate that there is no significant difference between different experimental groups. No data (ND) were obtained from group II (fish exposed to 5% untreated WSFO) and III (fish exposed to 10% untreated WSFO).

## Data Availability

The original contributions presented in this study are included in the article. Further inquiries can be directed to the corresponding author.
